# Interaction of the Antimicrobial Peptide Aurein 1.2 and Charged Lipid Bilayer

**DOI:** 10.1038/s41598-017-03795-6

**Published:** 2017-06-16

**Authors:** Durgesh K. Rai, Shuo Qian

**Affiliations:** 1 Biology & Soft Matter Division, Oak Ridge National Laboratory, Oak Ridge, TN 37831 USA; 2 Center for Structural Molecular Biology, Oak Ridge National Laboratory, Oak Ridge, TN 37831 USA

## Abstract

Aurein 1.2 is a potent antimicrobial peptide secreted by frog *Litoria aurea*. As a short membrane-active peptide with only 13 amino acids in sequence, it has been found to be residing on the surface of lipid bilayer and permeabilizing bacterial membranes at high concentration. However, the detail at the molecular level is largely unknown. In this study, we investigated the action of Aurein 1.2 in charged lipid bilayers composed of DMPC/DMPG. Oriented Circular Dichroism results showed that the peptide was on the surface of lipid bilayer regardless of the charged lipid ratio. Only at a very high peptide-to-lipid ratio (~1/10), the peptide became perpendicular to the bilayer, however no pore was detected by neutron in-plane scattering. To further understand how it interacted with charged lipid bilayers, we employed Small Angle Neutron Scattering to probe lipid distribution across bilayer leaflets in lipid vesicles. The results showed that Aurein 1.2 interacted strongly with negatively charged DMPG, causing strong asymmetry in lipid bilayer. At high concentration, while the vesicles were intact, we found additional structure feature on the bilayer. Our study provides a glimpse into how Aurein 1.2 disturbs anionic lipid-containing membranes without pore formation.

## Introduction

Antimicrobial peptides are innate first line of defense in all living organisms. They are found to be in a variety of different species of amphibians, insects and plants. Their broad spectrum against a wide range of bacteria, quick action and receptor-free nature make them promising new drugs in a world threatened by increasingly antibiotic-resistant pathogens. The current consensus is that most antimicrobial peptides are membrane-active, and they target bacterial cells due to the differences in membranes between host cells and intruding bacterial cells ^1^. The preferential association and disruption of bacterial cells enable them to kill intruding organisms. Potentially, they could be developed as anti-tumor and anti-viral agent due to their nature of selectively targeting specific membranes.

**Figure 1:**
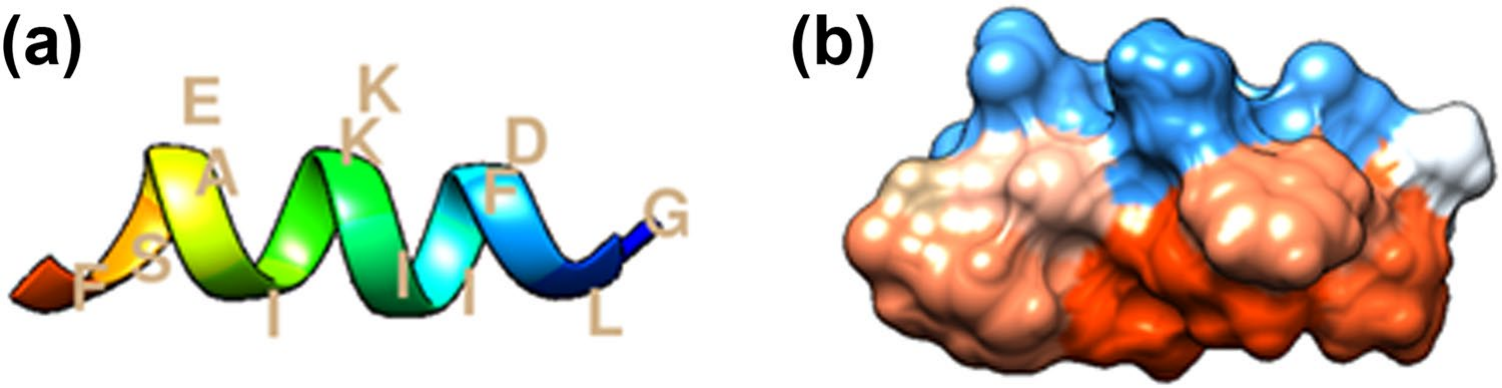
(**a**)A ribbon representation of aurein helical structure with amino acids labeled; (**b**) a side view of aurein hydrophobicity surface generated from PDB ID: 1VM5^40^ by UCSF Chimera^41^, blue represents hydrophilic surface and red represents hydrophobic surface.

Aurein peptides are a large family of peptides discovered in the skin secretions of *Litoria genus* of Australian frogs with various degree of antimicrobial activity. They are classified per their lengths. The peptide in our present study, Aurein 1.2 peptide (referred as aurein hereafter) is one of the shortest in the family with only 13 amino acids amidated at the C-terminal (Fig. 1a). Upon the discovery, it is known as one of the most potent antimicrobial peptides with a short sequence^2^. Through the action on membranes like many other antimicrobial peptides, it inhibits both intact Gram-negative and Gram-positive bacteria^3^. It also shows a moderate anti-cancer activity on 52 out of the 54 cancer cells in the NCI testing program, at the concentrations that can kill bacterial and cancer cells without harming mammalian cells^2^. Even more interestingly, it substantially enhances the endosomal escape of protein when conjugated with a cargo cationic protein^4^. This short yet effective membrane-active peptide is of great interest for a better understanding of the interaction between antimicrobial peptides and membranes. The understanding will help searching for effective therapeutic biomolecules or de novo design of novel antimicrobial agents. Indeed, another short peptide named LLAA, derived from human antimicrobial peptide LL-37 and identified as an aurein analog, has be found to have strong anti-HIV and antibacterial activities^5, 6^.

Structurally, aurein is amphipathic with well-defined hydrophobic and hydrophilic side along its helical axis (Fig. 1b). It is cationic and readily folds into α-helice in the presence of lipid membranes^2, 7^. It binds to model membranes with or without charged lipid but it is of higher affinity and more disruptive to the bilayer containing anionic lipids^8–10^. The interaction usually occurs on the surface of the membrane and causes disorder in chain and membrane thinning in a concentration dependent manner^7, 9, 11^. While many of aurein activities are similar to many other membrane-active antimicrobial peptides, there are conflicting reports on its membrane pore formation activity^7, 11, 12^. Many α-helical membrane-active antimicrobial peptides such as alamethicin, melittin, maganin are pore-forming peptides. They either form transmembrane pore through re-orienting in bilayer to line up a proteinous channel as described in “barrel-stave model”^13^ or in the form of peptide-lipid complex as in “toroidal model”^14, 15^ or “carpet model”^16^. In the present study, Oriented Circular Dichroism (OCD) afforded us to probe the orientation of aurein in bilayer^17^. Typically, antimicrobial peptide is found to be on the surface of lipid bilayer at low concentration and gets inserted into bilayer as its concentration increases^18^. The surface binding raises membrane tension. Over a threshold concentration, the orientation of peptide becomes perpendicular to the bilayer surface, a prerequisite for transmembrane pore formation. The stable transmembrane pore formation reduces membrane tension via changing from the peptide surface binding to the peptide pore binding^19^. Our OCD results indeed showed that aurein remained on the surface of the bilayer at low concentrations and became perpendicular to bilayer at a very high concentration without any transition state. Thereafter, we tried to detect the presence of transmembrane pore by using neutron in-plane scattering under the same condition, but found that there was no transmembrane water channel in the bilayer. Therefore, we conclude that since aurein doesn’t form transmembrane pores even while being perpendicular to the bilayer; it must disrupt the membrane by some other means.

Previous studies have shown that aurein disrupts membrane, especially in the presence of anionic lipids, however the structure and morphology change of lipid bilayers caused by aurein was largely unknown. For example, does a higher affinity to charged lipid mean that aurein is preferably associated with charged lipid? How does aurein modify charged or neutral lipid distribution at different charged lipid-to-neutral lipid ratios and different peptide concentrations? Here, we used Small Angle Neutron Scattering (SANS) to probe lipid bilayer structure of large unilamellar vesicle (LUV) with varying charged lipid ratios in the presence of aurein^20^. The lipid bilayer was a mixture of chain-deuterated neutral lipid (1,2-dimyristoyl(d54)-sn-glycero-3-phosphocholine, d54-DMPC) and protiated anionic lipid (1,2-dimyristoyl-sn-glycero-3-phospho-(1′-rac-glycerol), DMPG). With significant differences in scattering length density (SLD) between the chain-deuterated lipid and the protiated lipid, the distribution of lipid species in the inner leaflet and the outer leaflet can be investigated in detail. With SANS, we’ve found that some of the well-studied membrane-active peptides such as alamthicin and melittin are capable of modifying charged lipids or cholesterol distribution in lipid bilayers even at the low concentrations that are below their pore formation concentrations^20–22^. The technique affords us an opportunity to understand how this cationic short peptide influences the negatively charged lipid distribution in bilayers. We found that the more aurein was titrated into the LUVs solution, the more anionic DMPG was driven from the inner leaflet to the outer leaflet. At the same time, the bilayer thickness decreased as the peptide concentration increased, similar to other membrane-active peptides^19^. More interestingly, at high peptide concentration, an additional feature that had not been observed in other antimicrobial peptides (e.g. alamethicin, melittin^20^) started to appear along with a typical bilayer SANS profile. The feature corresponds to a structure of about 8 to 10 nm in size. Since the SANS from the LUVs solution was isotropic, we cannot decide whether the location of such structures was lateral heterogeneous segregation of the charged lipid and the neutral lipid or off the bilayer as extravesicular micelle-like budding. Nonetheless, our results suggest that aurein binding on the bilayer causes a strong redistribution of the charged lipid in the outer leaflet, and the formation of lipid-aurein complex. Such rearrangement makes membrane fragile and permeable, prone to further defect that is deadly. Such actions of aurein could well damage the membrane integrity without forming any transmembrane pore.

## Results and Discussions

### Orientation of Aurein in Bilayers

**Figure 2:**
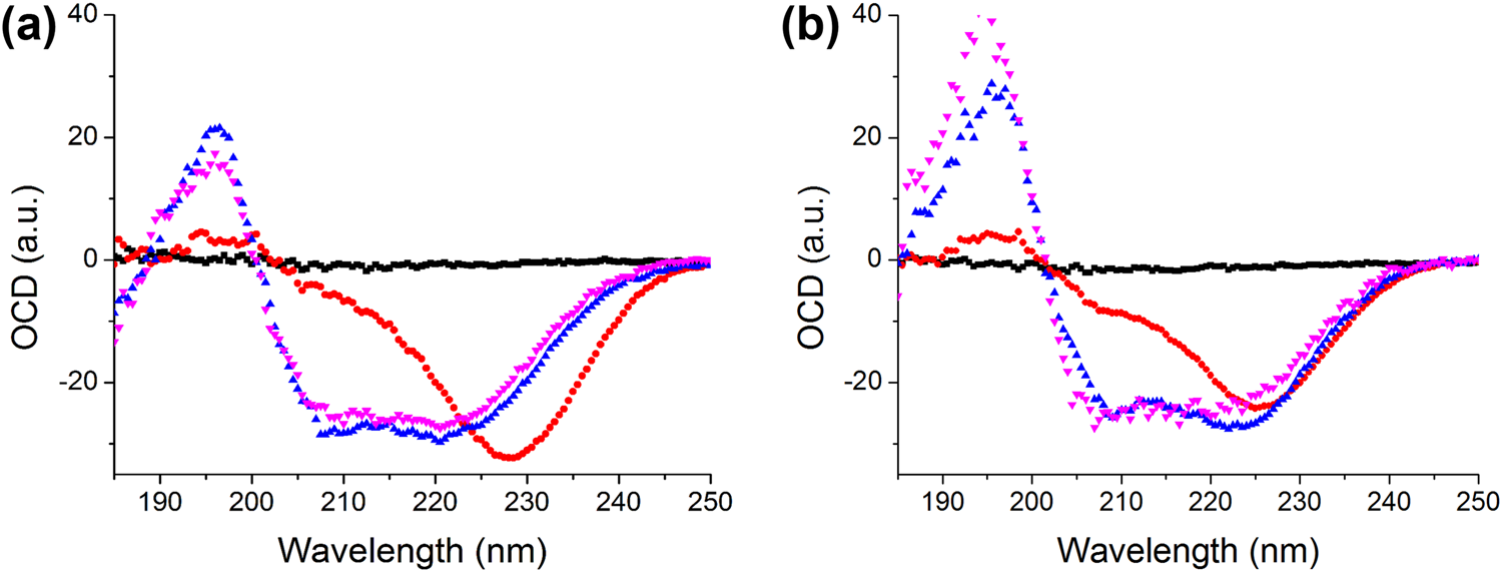
OCD measured at ~100% RH (**a**) DMPC: DMPG = 0.75:0.25; (**b**) DMPC: DMPG = 0.5:0.5. Lipid only (black); P/L = 1/100 (purple); P/L = 1/30 (blue); P/L = 1/10 (red).

A-helical membrane-active peptides usually exhibit distinctive circular dichroism spectra depending on the orientation of peptide in lipid bilayer^17, 18^. We performed OCD experiment on multilamellar samples under the relative humidity from ~20% to ~100% with two lipid compositions of different molar ratios of negatively charged lipid DMPC: DMPG = 0.75:0.25 and 0.5:0.5, respectively. For each lipid composition, a peptide-to-lipid ratio (P/L) series of P/L = 0 (lipid only), 1/100, 1/30, 1/10 were used. After normalizing to the peptide concentrations, we found that there were only two distinct spectra of aurein in the bilayer. At lower concentrations of P/L = 1/100 and 1/30, the spectra were similar in all lipid compositions with a positive peak at ~195 nm, and negative peaks at ~208 nm and ~220 nm. This indicates that aurein is lying on the surface of the bilayer, parallel to the bilayer regardless of the charged lipid ratio (S state, Fig. 2 purple and blue curves). At P/L = 1/10 for both lipid compositions, the spectra showed a sole but prominent negative peak near 225 nm, which pointed out that the peptide was oriented mostly perpendicular to the bilayer (I state, Fig. 2 red curves). The results are consistent with the OCD spectra shown by many other α-helical membrane-active peptides^17, 18^. However, what is absent are intermediate states transiting from S state to I state as the P/L increases. Many well-studied antimicrobial peptides such as alamethicin, melittin, etc. have been observed to undergo a gradual transition from S state to I state as peptide concentration increases^17, 19^. The intermediate states can be decomposed into a linear combination of S and I states to obtain the ratios of peptide inserted into a bilayer. In the case of aurein, it stays on the surface of bilayer with no spectrum change at all from P/L = 1/100 to as high as P/L = 1/30. It is only found to re-orient at P/L = 1/10, a relatively high concentration. The absence of any intermediate state in a moderate peptide concentration may be a sign of how this short peptide is accommodated by the lipid molecules. Even its hydrophobic and hydrophilic sides are well defined, its small length and small number of polar amino acids require a greater degree of cooperativity among nearby aurein peptides to overcome the energetics of reorientation in a bilayer. Individual or a small number of aurein may not be capable of inserting into a bilayer. This is compatible with previous report that of aurein forms oligomer to interact with the membrane in the MD simulation^9^ and that the dimerized aurein can disrupt giant unilamellar vesicles^7^. However, OCD is not able to detect the oligomerization state of aurein.

The DMPG ratio has a few minor effects on the spectra. In the I state (P/L = 1/10), the shifting of the negative peak from 228 nm in DMPC: DMPG = 0.75:0.25 to 225 nm in DMPC:DMPG = 0.5:0.5 suggests that the peptide, while being mostly perpendicular to the bilayer surface in the presence of more charged lipid, is slightly more titled^23^, indicating different association of peptide and lipid at different charge conditions. In the S state, the peptide is parallel to the bilayer surface. In samples with less charged lipid DMPC: DMPG = 0.75:0.25, the spectra from P/L = 100 and 1/30 are qualitatively identical, indicating that the peptide interacts with the bilayer indifferently even with a ~3-fold increase in concentration. At higher DMPG concentration (DMPC: DMPG = 0.5:0.5), a slight shift of the negative peak from ~220 nm to ~223 nm is a sign of varying peptide tilting on the bilayer surface. But the peptide remains mostly parallel to the bilayer with little sign of insertion. The minute difference in samples with different ratio of charged lipid shows that aurein is more active in the presence of DMPG, pointing toward a preferable association of aurein and anionic lipid compared to neutral lipid.

We also found the spectra didn’t change with relative humidity level from ~100% to ~20% (data not shown) at all P/L concentrations and both lipid compositions. Previously, we have found that varying water content in the sample could change peptide orientation, particularly for I state, as less water drives amphipathic peptide out of the bilayer into S state^13, 24^. The absence of such change suggests a stronger association of anionic lipid and aurein in I state.

### No Transmembrane Pore Formation

We examined the samples in which aurein was found to be perpendicular to the bilayer (P/L = 1/10) with neutron in-plane scattering to detect the presence of any transmembrane pore formation. The samples were made of protiated lipids and were hydrated with D_2_O. Any stable transmembrane pore formed in bilayer would be filled with D_2_O, resulting in large contrast in SLD between the water lumen and the rest of the lipid bilayer^25^. The scattering results of P/L = 1/10 in both lipid compositions (Fig. S1 in the Supplementary Information) were mostly flat, with only small humps around ~0.1 Å^−1^ due to oily streak defect in the multilayers. Therefore, we didn’t see any evidence of transmembrane pore formation even when aurein was found to be mostly perpendicular to the bilayer surface.

### Aurein Alters Charged Lipid Distribution

SANS experiment was performed on LUVs in solution. As in OCD and neutron in-plane scattering experiments, we used two lipid compositions with different charged lipid ratios: d54-DMPC: DMPG = 0.75:0.25 and d54-DMPC: DMPG = 0.5:0.5. The LUVs solution was dilute and free of inter-vesicle interaction, with lipid concentration of 2% (w/w) in 100% D_2_O, which has much lower incoherent neutron scattering background compared to H_2_O. The temperature was set to at 30 °C, above the gel-liquid phase transition temperature of both lipids. The chain-deuterated d54-DMPC delivers high neutron SLD contrast to the protiated chain of DMPG, therefore we can differentiate the distribution of two different lipids in bilayer. Aurein was titrated into the LUVs solution after the LUVs were extruded, about 12 hours prior to the SANS measurements to allow the samples to become equilibrium. Solution CD measurement showed aurein was mostly α-helical in the presence of LUVs (Fig. S2 in the Supplementary Information).

**Figure 3:**
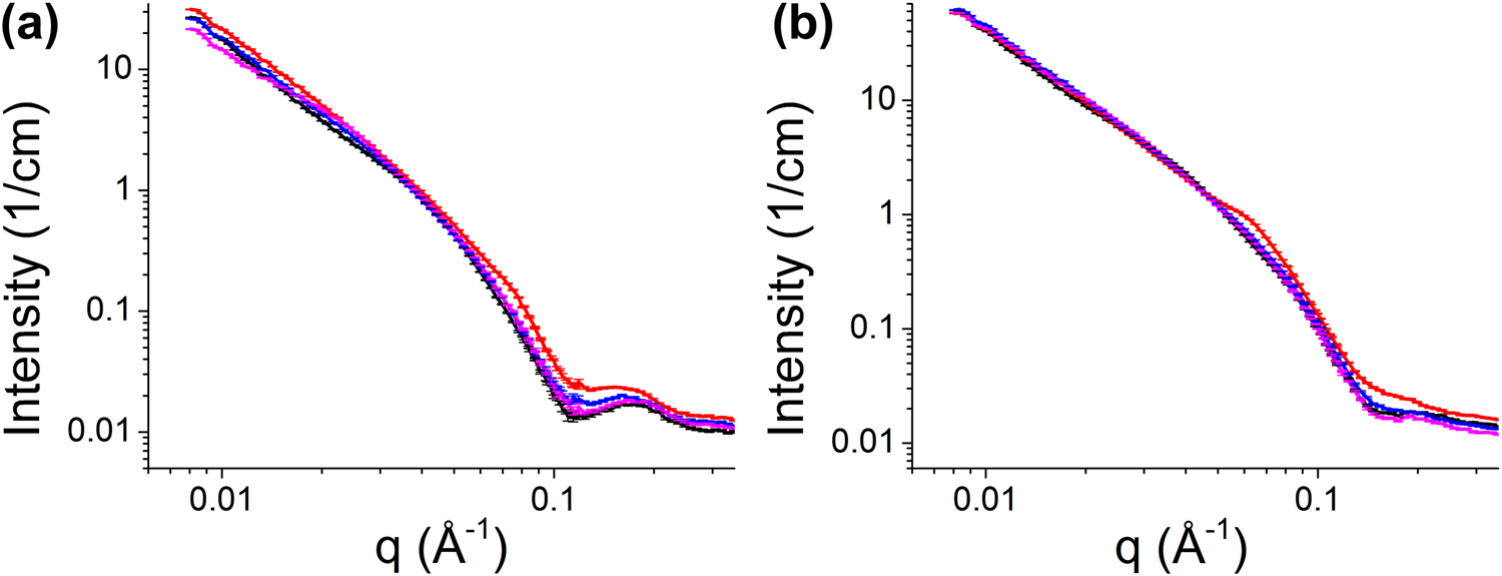
SANS curves in absolute scale (**a**) d54-DMPC: DMPG = 0.75:0.25 and (**b**) d54-DMPC: DMPG = 0.5:0.5 with various P/L ratios: lipid only (black), 1/100 (purple), 1/30 (blue), 1/10 (red).

The SANS experimental curves are shown in Fig. 3. In d54-DMPC/DMPG = 0.75:0.25 data (Fig. 3a), the intensity differences at the first minima near q = 0.1 Å^−1^ relative to the first peaks ~0.15 Å^−1^ can be seen diminishing with increasing peptide concentrations. At higher peptide concentrations, especially P/L = 1/10, the diminishing effect is so prominent that the dip seems to be disappearing. In d54-DMPC: DMPG = 0.5:0.5 data (Fig. 3b), the first minimum is already very shallow in lipid only sample. The same diminishing trend can be seen as the peptide concentration increases. At P/L = 1/10, the dip disappears with no apparent peak on the right, indicating a strong modification of bilayer structure.

For both lipid compositions, the scattering profiles from bilayer were dominant at all peptide concentrations, showing that the LUVs remained intact, which was confirmed by the dynamic light scattering. But in addition to the typical bilayer SANS profiles, other subtle features start to emerge in the curve: a small hump is around 0.075 Å^−1^ on the top of bilayer scattering in d54-DMPC: DMPG = 0.75:0.25 P/L = 1/10 sample. It is even more visible around 0.06 Å^−1^ in d54-DMPC: DMPG = 0.5:0.5 P/L = 1/10 sample.

**Figure 4:**
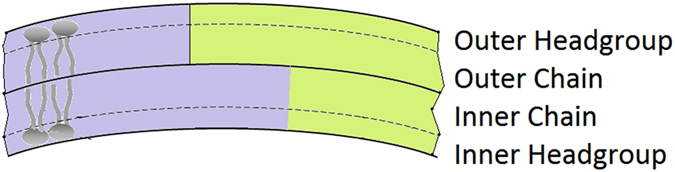
Four-layer lipid bilayer model used in the data fitting. Bilayer is divided into inner and outer leaflets with chain and headgroup regions. Blue and green represents different lipid compositions that are distributed unevenly in different leaflets.

**Figure 5:**
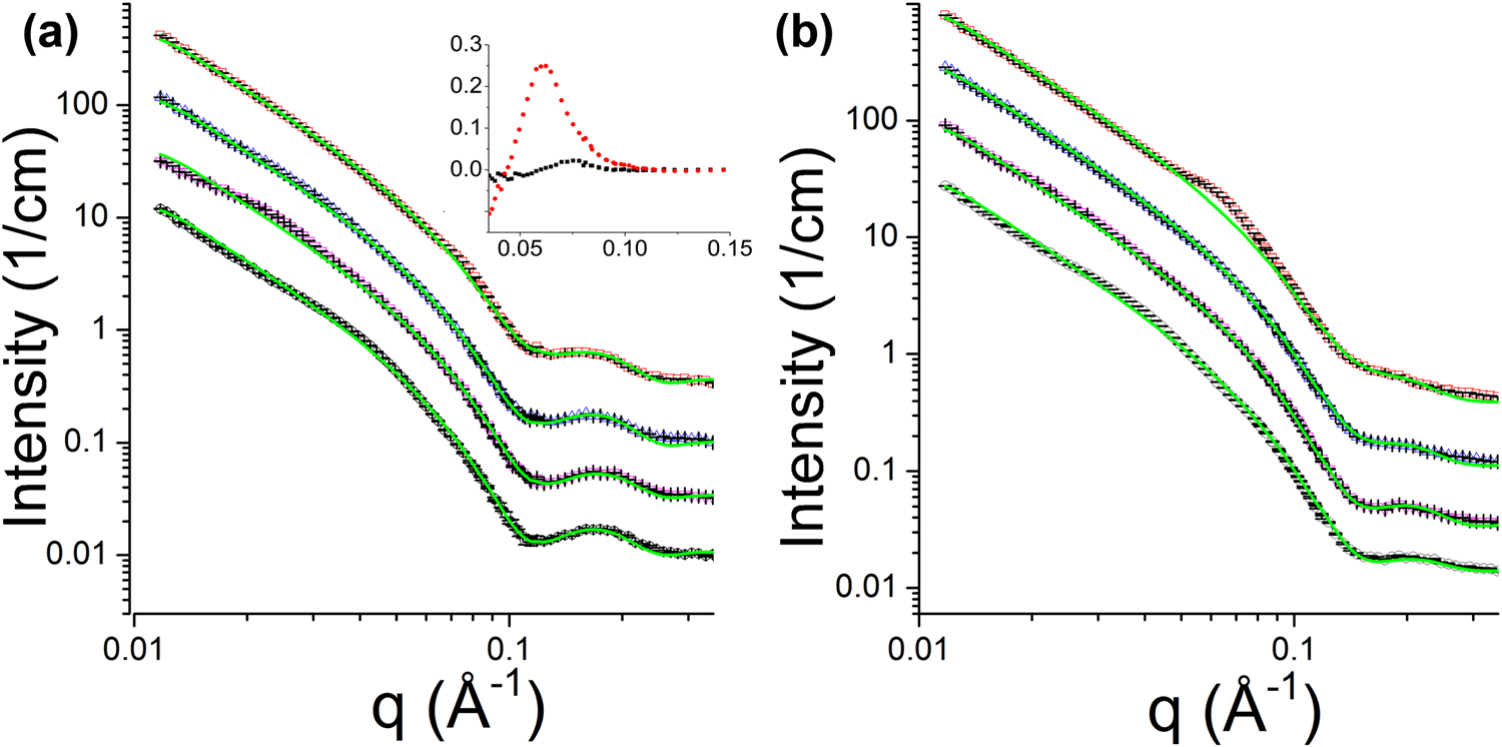
Model fitting results (green) overlaid with experimental data (from bottom to top: no peptide, P/L = 1/100, 1/30, 1/10). (**a**) d54-DMPC: DMPG = 0.75:0.25, (**b**) d54-DMPC: DMPG = 0.5:0.5. Curves are offset for clarity. The inset are subtractions of the model fitting curves from the experimental curves showing the humps in P/L = 1/10 samples (black: d54-DMPC: DMPG = 0.75:0.25, red: d54-DMPC: DMPG = 0.5:0.5).

To solve the structure of the bilayers, we employed a structural model which consists of relatively well-defined 4 layers of a lipid bilayer: inner leaflet headgroup, inner leaflet chain, outer leaflet chain, outer leaflet headgroup, as shown schematically in Fig. 4. For each layer, the neutron SLD is calculated from the lipid composition with specific ratios of d54-DMPC and DMPG. With such layers as shells, the neutron scattering intensity of LUVs is computed from the core-shell model. The fitting focuses on the *q*-range between 0.03 to 0.35 Å^−1^, as detailed in the Materials and Methods section, to emphasize on solving the bilayer structure. The model fitting results are shown in Fig. 5 and the parameters used in the fittings are in Table 1.

**Table 1:** Structural parameters obtained by model fitting.

d54-DMPC:DMPG	Inner Headgroup (Å)	Inner Chain (Å)	Outer Chain (Å)	Outer Headgroup (Å)	Total bilayer thickness (Å)	Nw	d54-DMPC ratio in the inner leaflet	Scale factor	background	Polydispersity
0.75:0.25 Lipid only	8.2	14.3	14.5	9.3	46.3 ± 1.0	8.0	81.6 ± 0.2%	0.012	0.0096	0.369
0.75:0.25 P/L = 1/100	8.3	14.5	14.0	9.0	45.8 ± 1.0	9.0	82.5 ± 0.2%	0.014	0.0104	0.384
0.75:0.25 P/L = 1/30	7.5	14.6	14.1	9.1	45.3 ± 1.0	6.4	85.0 ± 0.2%	0.012	0.0100	0.348
0.75:0.25 P/L = 1/10	6.8	14.0	14.0	9.5	44.3 ± 1.0	7.5	87.0 ± 0.3%	0.014	0.0119	0.329
0.5:0.5 Lipid only	8.0	13.5	11.7	9.3	42.5 ± 1.0	4.8	63.4 ± 0.2%	0.012	0.0136	0.300
0.5:0.5 P/L = 1/100	6.0	13.7	11.4	9.5	40.6 ± 1.0	1.5	66.3 ± 0.2%	0.011	0.0108	0.393
0.5:0.5 P/L = 1/30	6.3	13.5	12.7	9.3	41.8 ± 1.0	3.0	70.7 ± 0.2%	0.012	0.0116	0.300
0.5:0.5 P/L = 1/10	6.0	14.5	14.5	9.5	44.5 ± 2.5	5.0	71.8 ± 0.3%	0.014	0.0136	0.300

The total bilayer thickness is the sum of the head group and the chain thickness of both inner and outer leaflets. The thickness of various shells is correlated; therefore, the total bilayer thickness and its uncertainty are more meaningful parameters. N_w_ is the number of water associated per lipid head group.

The most notable result from the fitting is an uneven distribution of d54-DMPC and DMPG between the inner and the outer leaflets of the bilayer. From the inner leaflet DMPC ratio in Table 1, we can see that the lipids are unevenly distributed even without the presence of aurein. For example, in d54-DMPC: DMPG = 0.75:0.25 bilayer, if d54-DMPC and DMPG were distributed evenly across the bilayer, the d54-DMPC ratio and the DMPG ratio in both leaflets would be 75% and 25%, respectively. Instead, the d54-DMPC ratio within the inner leaflet of pure lipid sample is found to be 81.6%, indicating that the inner leaflet is less abundant in DMPG (100%−81.6% = 18.4%) with respect to the outer leaflet (DMPG ratio 50%−18.4% = 31.6%). Binding of more aurein to the vesicle exacerbates the asymmetry with a steady depreciation in DMPG of the inner leaflet., increasing the DMPC ratio up to 87% in the inner leaflet at P/L = 1/10. In d54-DMPC: DMPG = 0.5:0.5 bilayer, the asymmetry in the peptide free bilayer is very prominent: the DMPC ratio in the inner leaflet is 63.4% instead of the nominal 50%, likely due to higher overall charged lipid ratio in the sample. For the same reason, the asymmetry increases with peptide concentrations even more rapidly with the increment of ~3% in d54-DMPC: DMPG = 0.5:0.5 instead of ~2% in d54-DMPC: DMPG = 0.75:0.25 for the next higher P/L values. This, as well, points to a stronger interaction between aurein and DMPG. It is likely that not all peptide titrated into the LUVs solution binds to the vesicle bilayer. Higher charged lipid fraction in the vesicle bilayer can promote more peptide binding and interaction. Sequentially, more peptide binding augments the asymmetric distribution of DMPG between bilayer leaflets.

It is noteworthy that, in the SANS curves, the gradual elevation changes in the first minima relative to the peaks on the right is a hallmark of changing asymmetric lipid bilayer containing deuterated and protiated lipids. We have calculated a series of simulated SANS curves from bilayers with increasing asymmetry without changing other parameters (Fig. S3 in the Supplementary Information). The steadily diminishing of the first minima is quite remarkable.

In all the lipid compositions, the thinning of lipid bilayers caused by aurein is notable at various peptide concentrations, with the exception of d54-DMPC: DMPG = 0.5:0.5 P/L = 1/10 sample. Since the OCD has shown that the peptide is getting integrated into bilayer, this thinning effect caused by aurein is inevitable, like many other α-helical membrane-active peptides^19, 26^. It is a direct result of membrane tension caused by aurein’s binding on the surface. As for the exception of d54-DMPC: DMPG = 0.5:0.5 P/L = 1/10 sample, we attribute it to a challenging fit owing to the noticeable hump (near ~0.06 Å^−1^) caused by additional, probably more drastic change on the vesicle bilayer, which the core-shell model does not consider. The deviation in the fit around the hump is obvious, causing larger uncertainty in the fitting parameters.

To understand the additional structural feature in P/L = 1/10 samples, we subtracted the model fitting curves from the experimental curves (Fig. 5 inset). From the peak position, the sizes of the structure feature are ~84 Å (=2π/0.075 Å^−1^) in d54-DMPC: DMPG = 0.75:0.25 and ~105 Å (=2π/0.06 Å^−1^) in d54-DMPC: DMPG = 0.5:0.5. The sizes are about twice of a single bilayer thickness, suggesting that micelle-like lipid-peptide complex in adjacent to the vesicle bilayer is budding out as aurein strongly interacts with DMPG. However, because SANS data from LUVs solution is isotropic, we cannot explicitly determine the orientation of the structure to be lateral on the bilayer or off the bilayer plane. We cannot rule out lateral clustering of charged lipid either, as the size is in line with observed lipid domains in model LUVs^27^.

Another observation is that the numbers of water molecules in the model are generally smaller in d54-DMPC: DMPG = 0.5:0.5 samples than in d54-DMPC: DMPG = 0.75:0.25 samples. It is possible that the presence of more peptides in the bilayer replaces water in the vicinity because of the preferable aurein-DMPG interaction.

### Discussion on the Mode of Action

Previous studies have shown that many membrane-active antimicrobial peptides are able to form stable transmembrane pores in model lipid bilayers once the peptide concentration reaches a critical value^28^. It is known that the peptide binding on the surface increases membrane area expansion, and disorders headgroup and acyl chain^3^, which is proportional to membrane tension^19, 29^. The transmembrane pore formation can be explained by the need to reduce membrane tension due to peptide binding on the surface, as the peptide insertion lessens membrane tension. Therefore, forming transmembrane pore is a way of stabilizing the peptide-lipid complex in their interaction. This is especially true for amphipathic helical peptides with well-defined hydrophobic and hydrophilic sides^16^. In our results, we observed the thinning of the lipid bilayer thickness upon aurein binding at low peptide concentrations, and the orientation change of aurein at high P/L, yet no transmembrane pore was observed under such conditions. One notable feature of aurein is its short sequence. Compared to other typical pore forming peptides such as alamethicin (20 a.a.) and melittin (26 a.a.), the tension relieved from the binding energy to the pore energy by an aurein is significantly smaller than longer peptides. The condition for stable pore formation may not be satisfied by re-orienting and relocating aurein in the bilayer^19^. In addition, the preferential interaction between aurein and the charged lipid complicates the alteration in membrane tension. An inhomogeneous lipid bilayer correlated with the varying distribution of charged lipids may make the formation of stable pore further impossible. Hence the pore formation is not favorable even as aurein re-orients at high peptide concentration in the bilayer, as observed in our OCD and neutron in-plane scattering experiments.

Our results from SANS suggest that aurein has profound effects on charged lipids: when it binds to bilayer with negatively charged lipid, aurein significantly modifies the lipid distribution between leaflets. DMPG in the bilayer redistributes towards the outer leaflet, making the uneven distribution of charged and neutral lipid between leaflets even more significant. This is similar to other antimicrobial peptides we have studied before. For example, alamethicin enriches the outer leaflets with negatively charged DMPG^20^. Melittin likewise drives negatively charged DMPG and DMPS to the outer leaflets of the bilayer^20, 30^. These evidences point to a common mechanism of membrane-active antimicrobial peptide on modulating charged lipid in membrane. It is known that an appropriate presence of different lipids is important for many cell functions. For example, PG lipid is essential for guiding the signal transducing protein EIIA^Glc^, a protein that belongs to the phosphoenolpyruvate carbohydrate phosphotransferase system, to bind to the maltose transporter^31^. Another example is that negatively charged lipids, such as PG lipid, control the normal function of voltage-dependent K^+^ channel proteins in the membrane^32^. The modification of lipid distribution by antimicrobial peptides could have lethal effects on the normal function of bacterial membranes, when the balance of charged and neutral lipids across bilayer is disturbed or even destroyed^33, 34^.

The redistribution of charged lipids into one leaflet of a bilayer also promotes clustering of lipid. The increase in inhomogeneity across the bilayer may affect mechanical and transport properties of membranes, stiffen the bilayer, and cause membrane defect^35^. The clustering of charged lipids likewise becomes a preferable target for cationic peptides. The stronger interaction promoted by electrostatic interaction makes lipid bilayer prone to peptide binding and accumulation in the surrounding area of the charged lipid, causing further damage to the membranes. The humps observed in the SANS curve at high peptide concentration may be a consequence of this clustering. It is noteworthy that this is only observed for aurein, probably due to its short length, as we didn’t observe any hump in our studies with alamethicin and melittin^20, 21, 30^. It is possible that aurein, either in oligomeric or monomeric form, clusters or rounds up DMPG into the outer leaflet. After the initial electrostatic interaction, the hydrophobicity drives aurein to form complex with lipids, probably with mostly charged lipids. Once the complex is formed adjacent to the bilayer, the peptide orientation is not limited to the bilayer surface. Eventually with sufficient peptide binding and charged lipid redistribution, micelle-like aggregate may be formed, resulting in membrane mass loss and causing extensive damage to membranes. This hypothesis provides an alternate mode of action to transmembrane pore formation on how aurein interacts with membranes containing negatively charged lipids.

## Materials and Methods

### Materials

1,2-dimyristoyl-sn-glycero-3-phosphocholine (DMPC), 1,2-dimyristoyl(d54)-sn-glycero-3-phosphocholine (d54-DMPC) and 1,2-dimyristoyl-sn-glycero-3-phospho-(1′-rac-glycerol) (DMPG) were purchased from Avanti Polar Lipids, Inc. (Alabaster, AL, USA). Aurine 1.2 peptide (seq-CH) (>95% purity) was synthesized by GenScript USA Inc. (Piscataway, NJ, USA). All materials were used as delivered.

### Oriented Circular Dichroism

The stock solution for aurein was prepared by titrating peptide in a chloroform/TFE = 3/1 (v/v) mixture (C3T1) in 1 mg/ml concentration by weight. The stock solutions for lipids were prepared by dissolving DMPC and DMPG in molar ratios of 0.75: 0.25 and 0.5:0.5, respectively, in C3T1 solvent in 25 mg/ml concentration. The aurein stock, lipid stocks and the C3T1 solvents was then added in appropriate ratios to obtain P/L = 1/10, 1/30 and 1/100, respectively, in addition to lipid only samples. Thereafter the mixtures were slowly deposited onto 19 *mm* × 19 *mm* × 0.5 *mm* quartz substrates, and slowly dried for the C3T1 solvent to evaporate under ambient temperature and atmosphere in a fume hood. The film samples were formed after organic solvent evaporation then kept in vacuum overnight to ensure all organic residues were completely removed. The thin films on the quartz substrates were sealed in a jar with H_2_O and left for equilibration for ~4 hours at ~37 °C, in order to obtain equilibrated samples that were well rehydrated. The films looked uniform upon visual inspection with total lipid amount of ~1 mg for all samples. The samples are then placed into a relative humidity controlled chamber controlled by a humidity generator (InstruQuest Inc, Florida, USA), which provides desired relative humidity for samples during the OCD measurements.

The OCD measurements were collected by using a JASCO J-810 (JASCO, Tokyo, Japan). The CD spectra were recorded with a scan speed of 100 nm/min, 0.5 s response time, and a bandwidth of 1 nm in the range of 190–260 nm. Each spectrum was an average over 5 scans. The results were normalized roughly by the peptide concentration (P/L) in the sample.

### Neutron In-plane Sample

The multilamellar samples for in-plane scattering were mixed by a procedure similar to the OCD samples. Protiated DMPC and DMPG were used to provide good contrast between possible D_2_O filled lumen and the lipid bilayers. After the organic solvent was removed by vacuum, the sample mixtures were mixed with water and were frozen at −80 °C. After lyophilization removed trace of H_2_O, the samples were incubated with D_2_O for 24 hours. The fully hydrated gel-like samples looked clear and were sealed into a quartz cell assembly for neutron measurement. The neutron measurement was similar to the SANS measurement in terms of instrument configuration, as described in the next section.

### Small Angle Neutron Scattering

The LUVs were prepared by dissolving d54-DMPC and DMPG in the molar ratios of 0.75:0.25 and 0.5:0.5 in C3T1 solution which was dried under vacuum overnight to ensure complete removal of all organic residues. The lipid mixtures were then mixed with D_2_O to obtain 40 mg/ml stock solution and vortex mixed for ~10 minutes. The vortexed solutions were then exposed to a set of four freeze-thaw cycles by alternately placing the suspension in a warm water bath at 50 °C and a freezer at −80 °C. The final solution was again vortexed for ~10 minutes before extruding through a mini-extruder, preheated at ~45 °C for ~30 minutes. The extruder, purchased from Avanti Polar Lipids (Alabaster, AL, USA), was fitted with Whatman® Nuclepore™ Track-Etched membranes with an average pore diameter of 0.1 µm. The extrusion was performed in sets of 10 passes, to obtain the final LUVs solutions. Dynamic light scattering (Wyatt Technology Corp., Santa Barbara, CA, USA) was used to check the size and stability of LUVs.

The stock solution for aurein was prepared by dissolving it in D_2_O as peptide stock solution of 10 mg/ml. The final samples of 2% (w/w) lipid solution were prepared by titrating the 40 mg/ml lipid stock LUV solution with appropriate amount of aurein stock solution and D_2_O, to obtain P/L ratios of 1/10, 1/30, 1/100, in addition to lipid only solution. After ~24 hours of equilibrium, the final samples were filled into cylindrical quartz cells (Hellma, Germany) with 1 mm path lengths for SANS data collection.

SANS measurements for both LUVs and multilamellar in-plane samples were performed at the Bio-SANS instrument at the High-Flux Isotope Reactor (HFIR) of Oak Ridge National Laboratory^36^. For the Bio-SANS experiment, wavelength of neutron was set to 6 Å (Δλ/λ ~0.15). The instrument was configured to provide an effective *q*-range of ~0.01–0.4 Å^−1^. All SANS measurements were performed at 30 °C, well above the phase transition temperature of DMPC and DMPG (~23 °C). The SANS data from the position sensitive 2D detector was reduced to 1-D profiles I(*q*) vs. *q*, by using facility supplied data reduction software MantidPlot that corrects for detector sensitivity, instrument dark current, sample transmission and solvent background^37^.

### SANS Data Model and Fitting

There was no inter-particle interaction between LUVs at the dilute lipid concentration used in the experiment, therefore the scattering was only a representation of the bilayer structure with structure factor = 1. Since the lipid bilayer was relatively well defined along its normal direction, a core-multiple-shell model was used to represent the structure. From the water core of LUV outwards, the shells are inner leaflet headgroup, inner leaflet chain, outer leaflet chain, outer leaflet headgroup, respectively, as shown schematically in Fig. 4. The neutron scattering lengths and volumes of the various components used to calculate the neutron SLDs in the model are tabulated in Table 2.

**Table 2:** Volumes and neutron scattering lengths of the components.

Component	Volume (Å^−3^)	Scattering length (fm)
aurein	1792^42^	539.4
PC headgroup	320^43^	60.09
PG headgroup	300^44^	86.70
d-54 lipid chain	780^43^	533.16
lipid chain	780^43^	−29.06
D_2_O	30	19.14
H_2_O	30	−1.68

Neutron scattering lengths are calculated from Sears^45^.

The SLDs of the inner and outer leaflet headgroup of were calculated in the model by equation (1) and (2), respectively as

1 $${\rho }_{in,head}=\frac{({b}_{pc}{r}_{in,dmpc}+{b}_{pg}{r}_{in,dmpg})+{N}_{W}{b}_{D-buffer}+{r}_{binding}{r}_{in,peptide}{b}_{peptide}(P/L)}{({V}_{pc}{r}_{in,dmpc}+{V}_{pg}{r}_{in,dmpg})+{N}_{W}{V}_{D-buffer}+{r}_{binding}{r}_{in,peptide}{V}_{peptide}(P/L)}$$

2 $${\rho }_{out,head}=\frac{({b}_{pc}{r}_{out,dmpc}+{b}_{pg}{r}_{out,dmpg})+{N}_{W}{b}_{D-buffer}+{r}_{binding}{r}_{out,peptide}{b}_{peptide}(P/L)}{({V}_{pc}{r}_{out,dmpc}+{V}_{pg}{r}_{out,dmpg})+{N}_{W}{V}_{D-buffer}+{r}_{binding}{r}_{out,peptide}{V}_{peptide}(P/L)}$$

where, *r*_*in,dmpc*_, *r*_*in,dmpg*_, *r*_*out,dmpc*_ *r*_*out,dmpg*_ is the molar fraction of DMPC and DMPG in inner leaflet and outer leaflet, respectively. *b*_*pc*_ and *b*_*pg*_ are the SLDs of the PC and PG headgroups, respectively. *N*_*w*_ is the number of water molecules in the headgroup region of each leaflet; *b*_*D-buffer*_ is the SLD of the solution. The *V* are the corresponding molecular volumes of the respective components as noted in subscripts. The ratios of DMPC and DMPG in both inner and outer leaflet are related. For example, in d54-DMPC:DMPG = 0.75:0.25, for either leaflet *r*_*dmpg*_ = 100%-*r*_*dmpc*_, and the ratio of d54-DMPC in the inner leaflet is related to the outer leaflet by *r*_*in,dmpc*_= 150%-*r*_*out,dmpc*_ sample. Therefore, once the r_*in,dmp*_c is determined, the other three ratios can be derived. If the bilayer is homogenous between the two leaflets, *r*_*in,dmpg*_ should be 25% with *r*_*in-dmpc*_=75%; *r*_*out,dmpc*_=75%, *r*_*out,dmpg*_ = 25%. A deviation from these values implies an asymmetric distribution of the two lipid components between the inner and outer leaflets of the bilayer. r_*bindin*g_ is the peptide binding ratio to the lipid bilayer; r_*in,peptid*e_ and r_*out,peptid*e_ are peptide distribution ratio in the inner and outer bilayer, respectively. *P/L* is peptide-to-lipid ratio. Practically, the contribution of peptide to the scattering intensity, even at the highest P/L ratio, is insignificant due to dominated LUVs scattering. From the computed intensity with model SLD calculated from equation 1 and 2, the results changed little with or without accounting the peptide parameters, therefore, the peptide parameters are negligible in the equations. Hence, r_*binding*_ was assumed to be 1, with r_o*ut,peptid*e_ = 1 and r_i*n,peptid*e_ = 0 assuming all peptides were located in the outer leaflet.

The SLDs of the chain region were given by

3 $${\rho }_{in,chain}=\frac{({b}_{dmpc,chain}{r}_{in,dmpc}+{b}_{dmpg,chain}{r}_{in,dmpg})}{({V}_{dmpc,chain}{r}_{in,dmpc}+{V}_{dmpg,chain}{r}_{in,dmpg})}$$

4 $${\rho }_{out,chain}=\frac{({b}_{dmpc,chain}{r}_{out,dmpc}+{b}_{dmpg,chain}{r}_{out,dmpg})}{({V}_{dmpc,chain}{r}_{out,dmpc}+{V}_{dmpg,chain}{r}_{out,dmpg})}$$

The scattering intensity from the core-shell model was calculated by:

5 $$I(q)=C{[\sum _{i=1}^{5}\frac{3{V}_{i}({\rho }_{i}-{\rho }_{i-1}){j}_{1}(q{d}_{i})}{q{d}_{i}}]}^{2}+bg$$

In the equation, *C* and *bg* are a scale factor and a constant background, respectively. ${\rho }_{i}$ are the SLDs of each shell, and ${\rho }_{0}$ is the neutron SLD of the core, which is equal to that of the D_2_O solution. *V*_*i*_ is the volume of each shell and *j*_1_(*x*) is the Bessel function. The *d*_*i*_ (i = 1, 2, 3, 4, 5) are the radii of the core and thicknesses of shells in the model, respectively. d_1_ is set to 50 nm as determined by lipid extrusion membrane filter pore size and dynamic light scattering. The variations in d_1_ only affect the scale factor for the intensity.

The computed intensity was convoluted with instrument resolution function^20^ and the polydispersity of the vesicle size distribution approximated by the Schulz distribution^38^.

The goodness of fit is evaluated by a chi-square function defined as

6 $${\chi }_{q}^{2}=\frac{1}{N-p}{\sum _{i=1}^{N}[\frac{{I}_{\mathrm{mod}el}(q)-{I}_{\exp }(q)}{{\sigma }_{\exp }(q)}]}^{2}$$

where N is number of data points in a given *q*-range, *p* is number of parameters in the model.

The structure probed in the studies is the lipid bilayer; hence the search is focused on *q*-range between 0.05 Å^−1^ and 0.35 Å^−1^. The fitting parameters are constrained by reasonable ranges of DMPC ratios r_in,dmpc_, the thickness of headgroups and chain regions as well as their total thickness. The coarse search went through all possible combinations of parameters with a step size of 0.5 Å for the thicknesses, 2% for r_in,dmpc_. At each step, *lsqcurvefit* algorithm in MATLAB (MathWorks, Boston, MA, USA) was used to determine best fitting parameters that were not constrained: scale factor, constant background and polydispersity in the *q*-range of 0.075 Å^−1^ to 0.35 Å^−1^. Then for each set of the parameters, χ^2^ for different q ranges were calculated to evaluate the goodness of fit on different regions of SANS curves: 0.08 Å^−1^–0.19 Å^−1^ for the first minimum; 0.05 Å^−1^–0.35 Å^−1^ for the overall peak. From 10 best results that scored with the least χ^2^ in both regions, a set of consensus parameters was obtained by averaging. Afterwards, a manual fine adjustment was performed within the coarse steps to find the best fits that minimize χ^2^ in various *q*-ranges mentioned above. The uncertainties were estimated from the manual adjustment that at least double χ^2^ in the q-range of the first minimum 0.08 Å^−1^–0.19 Å^−1^. The thicknesses of shells are correlated, therefore the total bilayer thickness and its uncertainty are more meaningful.

The experiment was performed with 100% D_2_O contrast condition, which provides the lowest incoherent background for the system. The quality of the data, especially the subtle changes around the peak positions provide robust features for the model fittings. While more contrast variation data in different ratios of D_2_O and H_2_O might improve the fitting, any addition of H_2_O would diminish the weak features around between 0.08 Å^−1^ and 0.19 Å^−1^ significantly, which is important in solving the bilayer structure^20, 21, 30^. The overall bilayer structure is well constrained by a number of previous studies, and the results are consistent with our previous studies with or without contrast variation^20, 30, 39^.

## Electronic supplementary material

Supplementary Information for Interaction of the Antimicrobial Peptide Aurein 1.2 and Charged Lipid Bilayer
