# Supplementary material for: Interaction of the Antimicrobial Peptide Aurein 1.2 and Charged Lipid Bilayer
Source: Sci Rep. 2017 Jun 16;7:3719. doi: 10.1038/s41598-017-03795-6 (PMC5473820; doi:10.1038/s41598-017-03795-6)
Supplement: Supplementary file 1 — Supplementary Information for Interaction of the Antimicrobial Peptide Aurein 1.2 and Charged Lipid Bilayer [file 41598_2017_3795_MOESM1_ESM.pdf]

Supplementary Information for:

Interaction of the Antimicrobial Peptide Aurein 1.2 and Charged Lipid Bilayer

Durgesh K. Rai [1], Shuo Qian [1,2]*

[1]Biology & Soft Matter Division and [2]Center for Structural Molecular Biology, Oak Ridge National Laboratory, Oak Ridge, TN 37831, U.S.A.

Corresponding Author

* Shuo Qian; Oak Ridge National Laboratory; PO Box 2008; MS-6393; Oak Ridge, TN 37831;

email: qians@ornl.gov; phone: 865-241-1934

Notes

The authors declare no competing financial interests.

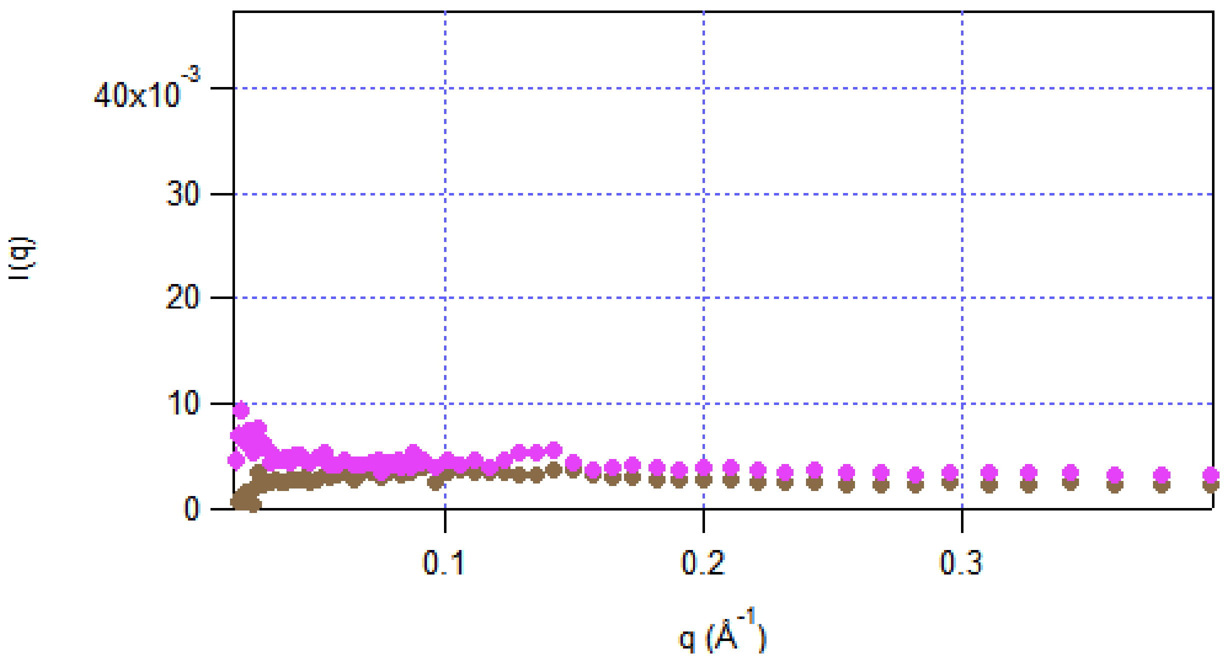

Figure S1 Neutron in-plane scattering measured from DMPC: DMPG= 0.75:0.25 (purple) and DMPC:DMPG = 0.5:0.5 (brown) at P/L=1/10

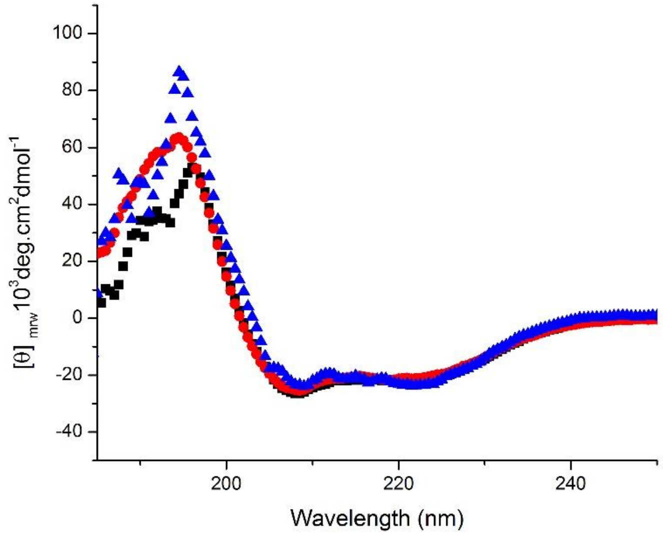

A

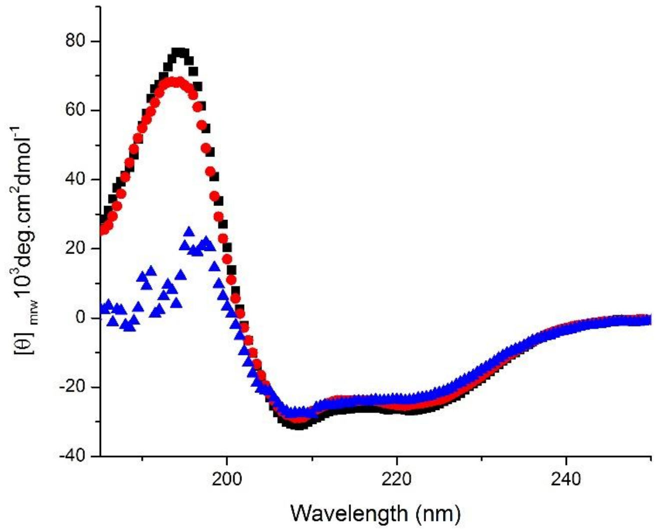

B

Figure S2. Circular dichroism of aurein in LUVs (A) DMPC:DMPG= 0.75:0.25 and (B) DMPC:DMPG = 0.5:0.5 at P/L=1/10 (black), 1/30 (red), 1/10 (blue)

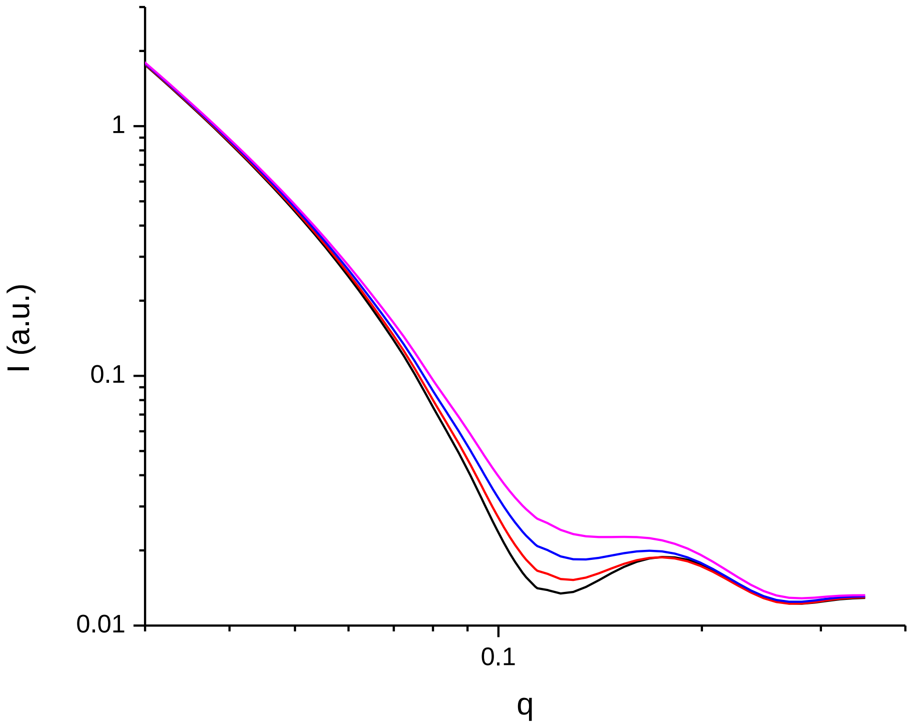

Figure S3 Curves computed from models to show the effect of increasingly asymmetric bilayer structure with a binary lipid composition. The asymmetry across bilayer leaflets increases from black to purple.
